# Boolean genetic network model for the control of *C. elegans *early embryonic cell cycles

**DOI:** 10.1186/1475-925X-12-S1-S1

**Published:** 2013-12-09

**Authors:** Xiaotai Huang, Long Chen, Hung Chim, Leanne Lai Hang Chan, Zhongying Zhao, Hong Yan

**Affiliations:** 1 Department of Electronic Engineering, City University of Hong Kong, Tat Chee Avenue, Kowloon, Hong Kong; 2 Department of Biology, Faculty of Science, Hong Kong Baptist University, Hong Kong

## Abstract

**Background:**

In *Caenorhabditis elegans *early embryo, cell cycles only have two phases: DNA synthesis and mitosis, which are different from the typical 4-phase cell cycle. Modeling this cell-cycle process into network can fill up the gap in *C. elegans *cell-cycle study and provide a thorough understanding on the cell-cycle regulations and progressions at the network level.

**Methods:**

In this paper, *C. elegans *early embryonic cell-cycle network has been constructed based on the knowledge of key regulators and their interactions from literature studies. A discrete dynamical Boolean model has been applied in computer simulations to study dynamical properties of this network. The cell-cycle network is compared with random networks and tested under several perturbations to analyze its robustness. To investigate whether our proposed network could explain biological experiment results, we have also compared the network simulation results with gene knock down experiment data.

**Results:**

With the Boolean model, this study showed that the cell-cycle network was stable with a set of attractors (fixed points). A biological pathway was observed in the simulation, which corresponded to a whole cell-cycle progression. The *C. elegans *network was significantly robust when compared with random networks of the same size because there were less attractors and larger basins than random networks. Moreover, the network was also robust under perturbations with no significant change of the basin size. In addition, the smaller number of attractors and the shorter biological pathway from gene knock down network simulation interpreted the shorter cell-cycle lengths in mutant from the RNAi gene knock down experiment data. Hence, we demonstrated that the results in network simulation could be verified by the RNAi gene knock down experiment data.

**Conclusions:**

A *C. elegans *early embryonic cell cycles network was constructed and its properties were analyzed and compared with those of random networks. Computer simulation results provided biologically meaningful interpretations of RNAi gene knock down experiment data.

## Background

Biological networks have been studied extensively in recent decades. They are useful to understand how genes and their interactions determine the functional organization in the cell. In *C. elegans*, several networks have been constructed so far, such as, protein-protein interaction (PPI) networks, genetic interaction (GI) networks, phenotypic networks, transcriptional regulatory networks, post-transcriptional regulatory networks, and other integrative networks [1]. However, the cell-cycle network of *C. elegans *has not been reported although such networks or models have been already constructed for other species, such as, the cell-cycle network of the budding yeast [2], the cell-cycle network model of the fission yeast [3], a Boolean model for the control of the mammalian cell-cycle [4], and mammalian cancer cell network during G1/S transition (MGSTR network) [5].

Living organisms develop from one cell (zygote) to many adult cells including many rounds of cell divisions. In each division, cells undergo sequential events, which regulate one cell to split into two daughter cells. These ordered series of events consist of cell-cycles, which is pervasively carried out in most species. Generally, cell-cycle includes four different phases: G1 (Gap 1), S (Synthesis), G2 (Gap2), and M phase (Mitosis) [6]. The M phase is a process of mitosis where cells stop growing at this stage and divide into two daughter cells. The other three phases, G1, S and G2, belong to the interphase. Particularly, the S phase plays the role of DNA synthesis by duplicating genome in nuclei. G1 and G2 phases are "Gap" phases, in which the G1 phase connects the end of the M phase of last cell-cycle to the beginning of the S phase in present cell-cycle, while the G2 phase ensures cell entering into the M phase correctly. Moreover, this mechanism is controlled by several regulators, which are able to interact with each other to achieve complex regulatory functions.

To model the biological processes, differential equation is commonly used which could be applied to biological pathway modeling and complex networks modeling [3]. This method provides more information on time evolution of the system [3]. However, timing is not a key factor in some robust designed biological networks [3]. Therefore, for the simplicity of computation, Boolean function, which possesses less parameter, has been used in some previous cell-cycle network models [2-5]. The variables in those models are Boolean type, which can take the value of 0 or 1, representing genes or proteins that are active or inactive respectively. This idea is attributed to the bistability of molecules, which means genes or proteins can switch in a Boolean manner in a biological system [7]. Many molecular regulatory factors possess the binary property. Boolean switches can also be observed frequently in some molecular circuits [8]. Although varying multistable behaviors exist in a biological system, it is found that bistability is an optimal regime to describe the state of genes and proteins [9]. To study the dynamics in genetic regulatory networks, a simple Boolean model is used to simulate the cell-cycle process. The main objective of the Boolean functions is to update the state of each node in the network as a function of time. For each node, their values at next time point are determined by the values of interacting nodes at present. The Boolean model is simple yet precise to describe dynamic properties of the network. Despite the simplicity, the Boolean model could indeed provide meaningful representation of the dynamics of the cell-cycle networks [2-5]. In our research, we study whether this Boolean model can describe dynamic properties of our proposed *C. elegans *early embryonic cell cycles network well and whether the *C. elegans *early embryonic cell cycles network is robust under noise conditions.

## Methods

### The *C. elegans *early embryonic cell cycle network

**Figure 1:**
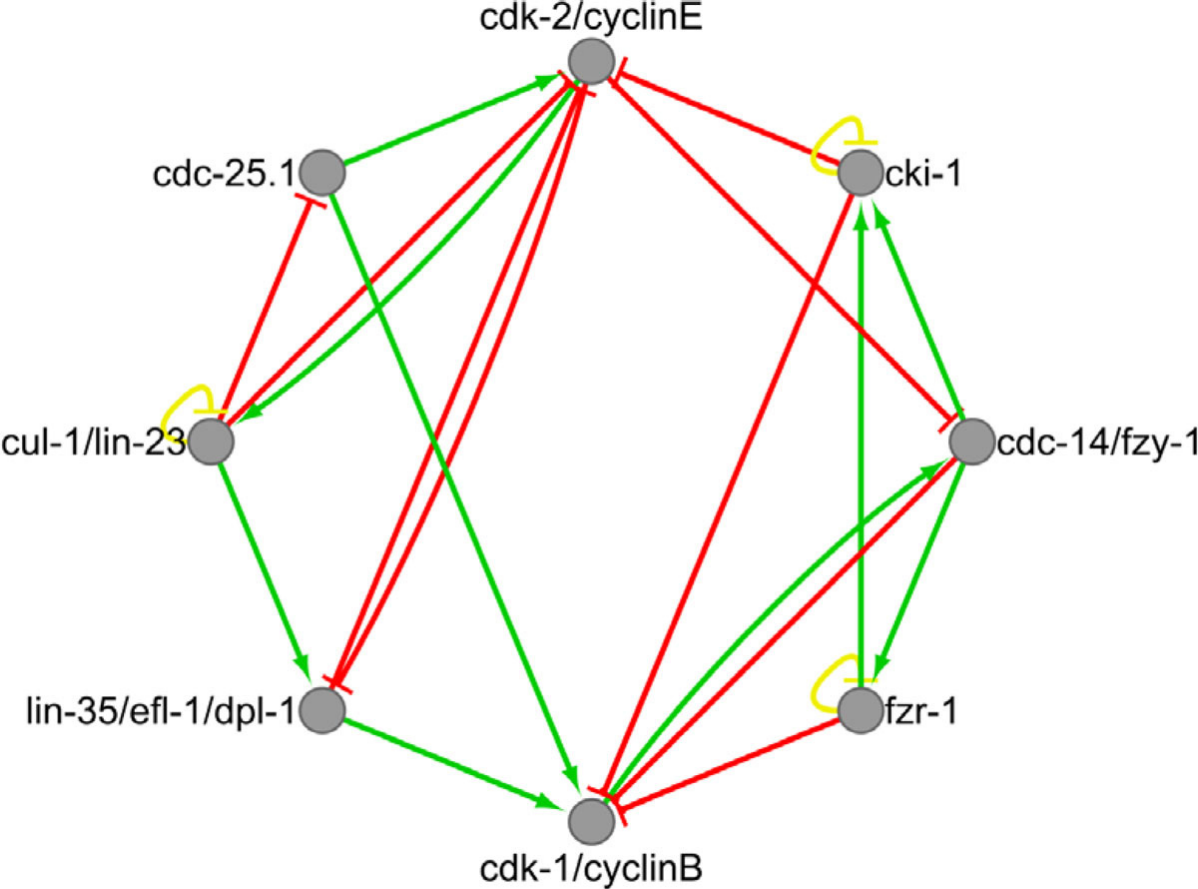
**The *C. elegans *early embryonic cell cycles network**. Each node represents a regulator in cell cycles. Green arrows and red edges represent 'activate' and 'repress' respectively. Yellow loop is a self-degradation for that node.

In *C. elegans *late embryo and larval stages, typical 4-phase cell cycles progress in body development and cell proliferation [6]. However, in early embryo, cell cycles progress by oscillations between S and M phases due to a rapid proliferation in cell numbers [6]. Currently there are about 600 genes related to cell-cycles in *C. elegans *reported in the Gene Ontology database. The core regulatory mechanism is related to the activity of complexes of CDKs (cyclin-dependent kinase) and cyclins. Specific CDKs and cyclins are responsible for controlling cells entering into or exiting from cell-cycle phases. Activation, repression, and degradation of CDKs and cyclins should also be considered. Based on literature studies of molecular regulatory interactions among the key regulators, we have constructed a Boolean genetic network model for the control of *C. elegans *early embryonic cell cycles, as shown in Figure 1. Interactions among nodes, corresponding references and descriptions are shown in Table 1.

**Table 1:** The rules of interactions in the *C.elegans *early embryonic cell cycles network.

Effector	Effected	Activation	Inhibition
*cdc-14/fzy-1*	*cdk-1*/cyclinB		*fzy-1 *is able to activate the APC (anaphase-promoting complex), which is for cyclin B degradation. -1 [10]
*cdc-14/fzy-1*	*fzr-1*	*fzy-1 *and *fzr-1 *encode orthologous proteins to Cdc20 and Cdh1 respectively in *S. cerevisiae*. The Interaction is inferred from the yeast cell-cycle network. 1 [2]	
*cdc-14/fzy-1*	*cki-1*	*cdc-14 *upregulates *cki-1 *for accumulation. 1 [11]	
*cdc-25.1*	*cdk-2*/cyclinE	*cdc-25.1 *activates *cdk-2 *by dephosphorylation. 1 [12]	
*cdc-25.1*	*cdk-1*/cyclinB	*cdc-25.1 *activates *cdk-1 *by dephosphorylation. 1 [12]	
*cdk-1*/cyclinB	*cdc-14/fzy-1*	Cyclin B and *cdc-14 *are orthologous to Clb1,2 and Cdc14, respectively. Interaction inferred from the yeast cell-cycle network. 1 [2]	
*cdk-2*/cyclinE	*cul-1/lin-23*	*cul-1 *and *lin-23 *encode a Skp1-Cul1-F box (SCF) protein complex. SCF will be turned on for cyclin E degradation when exiting from S phase. Here we infer that *cdk-2*/cyclinE triggers SCF. 1 [13]	
*cdk-2*/cyclinE	*lin-35/efl-1/dpl-1*		Inhibits -1 [11]
*cdk-2*/cyclinE	*cdc-14/fzy-1*		Cyclin E suppresses the expression of *cdc-14*. -1 [14]
*cki-1*	*cdk-2*/cyclinE		*cki-1 *encodes CDK inhibitory proteins which is rate limiting for S phase entry. -1 [13]
*cki-1*	*cdk-1*/cyclinB		*cki-1 *encodes CDK inhibitory proteins. Inhibit activity of *cdk-1*. -1 [15]
*cki-1*	*cki-1*		Add a self-degradation due to no inhibitory interaction on this node. This method was used in Li's model. -1 [2]
*cul-1/lin-23*	*cdk-2*/cyclinE		*cul-1 *and *lin-23 *encode a Skp1-Cul1-F box (SCF) protein complex for cyclin E degradation. -1 [13]
*cul-1/lin-23*	*cdc-25.1*		*lin-23 *negatively regulates the abundance of *cdc-25.1*. -1 [16]
*cul-1/lin-23*	*cul-1/lin-23*		Add self-degradation. -1
*cul-1/lin-23*	*lin-35/efl-1/dpl-1*	A synthetic interaction between *lin-23 *and *lin-35*. 1 [17]	
*fzr-1*	*cdk-1*/cyclinB		Loss of *fzr-1 *will decrease APC which is for cyclin B degradation. -1 [17]
*fzr-1*	*fzr-1*		Add self-degradation. -1
*fzr-1*	*cki-1*	*fzr-1 *promotes accumulation of *cki-1*. 1 [13]	
*lin-35/efl-1/dpl-1*	*cdk-2*/cyclinE		*lin-35 *negatively regulates *cye-1*. -1[11]
*lin-35/efl-1/dpl-1*	*cdk-1*/cyclinB	*efl-1/dpl-1 *promotes expression of cyclin B. 1 [18]	

Columns 1 and 2 represent the parent nodes and daughter nodes in their interactions. 1 or -1 after the descriptions in column 3 and 4 represent the weights for that interaction.

There are 8 nodes in the network, which are CDK/cyclin complex, inhibitors, and degraders. We combine several genes or proteins into one node based on their biological functions. The *cdk-2 *and cyclin E protein families are merged into one node since their complexes regulate the S phase entry and progression [13,19]. *Cdc-25.1 *encodes a phosphatase of the Cdc25 family, which activates CDKs by dephosphorylation [13,19]. *Cul-1 *and *lin-23 *encode proteins to form a Skp1-Cul1-F box (SCF) protein complex for cyclins degradation [13]. *Lin-35*, *efl-1 *and *dpl-1 *encode the tumor suppressor pRb and transcription factor E2F family, which form the Rb/E2F pathway for cell-cycle control [13]. *Cdk-1 *and cyclin B complexes promote the M phase entry and progression in *C. elegans *cell-cycle [13,19]. *Cki-1 *encodes a type of Cyclin-dependent Kinase Inhibitors pervasively exist from yeast to metazoan. CKIs act to inhibit cell-cycle progression. They are rate limiting for the S phase entry in *C. elegans *[13]. *Fzy-1 *and *fzr-1 *are substrates to the APC (anaphase-promoting complex) [13]. Therefore, the expression of *fzy-1 *and *fzr-1 *controls the activity of APC, whose function degrades the cyclin B family for the M phase exit [13]. *Cdc-14 *plays a parallel role to Rb/E2F pathway in *C. elegans *cell-cycle, which positively regulates the activity of *cki-1 *to inhibit entry into the S phase [13]. *Fzy-1*, *fzr-1 *and *cdc-14 *encode orthologous proteins to Cdc20, Cdh1 and Cdc14 respectively in *S. cerevisiae *[2,13]. Here, we merge *cdc-14 *and *fzy-1 *into one node because Cdc20 degrades an inhibitor of Cdc14 in *Saccharomyces cerevisiae *[2].

In Figure 1, red arrows represent deactivation, which includes inhibition, repression, or degradation, while green arrows represent activation. We also add self-degradation (yellow loops) for nodes, *cul-1/lin-23*, *fzr-1 *and *cki-1*, which are not repressed by others, based on the method of Li et al. [2]. The cell-cycle begins when *cdk-2*/cyclinE is turned on, which means cells enter into the S phase. Then *cul-1/lin-23 *is triggered to degrade the cyclinE family for the S phase exit. *Cul-1/lin-23 *activates *lin-35/efl-1/dpl-1*, which inhibits *cdk-2*/cyclinE. *Efl-1/dpl-1 *promotes expression of cyclin B, which represents the M phase entry. *Cdc-14/fzy-1 *and *fzr-1 *is triggered to up regulate the APC for cyclinB family degradation. At this stage, *cki-1 *is also activated for the inhibition of both *cdk-2*/cyclinE and *cdk-1*/cyclinB. Thus, cells start at entering into the S phase and end at exiting from the M phase, and wait for signals to enter into the next round of cell cycle.

The network and dynamic trajectories presented in this paper are obtained using Cytoscape [20].

### Dynamic model of the *C. elegans *early embryonic cell cycles network

Based on whether genes are expressed or not in a biological system, we assume that every variable (node), in the network, will take a Boolean value. That means every node has two possible states (on/off), which represents the activity of gene/protein. (1 represents 'on/active' and 0 represents 'off/inactive'). For each Boolean variable, its value at next time point is determined by all interacting nodes at the present time point via Boolean functions as follows:

(1) $$S_{i}\left(t+1\right)=\left\{\begin{array}{c} 1,\;\;\;\;\;\;\;\;\;\;\;\;\;\;\;\;\;\;\;\;\underset{j}{\sum }w_{ij}S_{j}\left(t\right)>\theta_{i}\\ 0,\;\;\;\;\;\;\;\;\;\;\;\;\;\;\;\;\;\;\;\;\underset{j}{\sum }w_{ij}S_{j}\left(t\right)<\theta_{i}\\ S_{i}\left(t\right)\;\;\;\;\;\;\;\;\;\;\;\;\;\;\;\;\;\underset{j}{\sum }w_{ij}S_{j}\left(t\right)=\theta_{i} \end{array}\right)$$

The update rule.

where $w_{ij}$ represents the weights for input edges from node  j to node  i, $S_{i}\left(t\right)$ denotes a state $\;\left(S\right)$ of node  i at any time  t, and t+1 represents the next time point. The threshold, which is denoted by $\theta_{i}$, is set to zero as default. The expression, $w_{ij}=1\;\text{or}\;-1$, represents activation or inhibition between interacted nodes. The weight for self-degradation is set to $w_{ii}=-1$. This Boolean model is an ideal model for real gene regulatory network in *C. elegans *early embryonic cell cycles due to its binary properties. Moreover, it also discovers the dynamic properties of the network based on its topological structure.

### RNAi gene knock down experiment data

The RNAi gene knock down experiment data were produced in our biology laboratory. Leica SP5 fluorescent confocal microscope was used to record the embryonic development of *C. elegans *from two of four cell stages. The cell-cycle lengths were extracted from their records. The detailed experiment methods were reported earlier [21,22]. The wild type and genes knock down of *cki-1*, *efl-1 *and *cdc-14 *experiment data are available in the supplementary files of this paper.

## Results

### Simulation of the *C. elegans *early embryonic cell cycle network

To study the dynamical properties of the *C. elegans *early embryonic cell cycle network, we simulated the changing of each gene or protein's value in the network by the Booleans functions at different time points. Since genes switch between expressed and non-expressed state in a biological system, each node has the same probability to be either 0 or 1, which represents its two possible states, namely off or on. In the *C. elegans *network, there were 2^8^=256 possible initial states (8 nodes), in the state space.

For each initial state, the update rules computed the value of each node at the next time point simultaneously. After several iterations, the state of the network would reach a stable state, which was called an attractor or a fixed point. The number of initial states that converged to an attractor was called the basin size (B) of this attractor. In our simulation, we found that all initial states would converge to five different attractors, which represented the dynamical results of the *C. elegans *network model. Moreover, it was observed that most initial states would converge to the largest attractor, where the basin size was found to be 219 or 85.5% of the state space (Table 2). This result showed that the *C. elegans *early embryonic cell cycles network was robust under different states of genes or proteins.

**Table 2:** Basin size of fixed points and their corresponding network states.

Basin size	*cdk-2*/cyclinE	*cdc-25.1*	*cul-1/lin-23*	*lin-35/efl-1/dpl-1*	*cdk-1*/cyclinB	*fzr-1*	*cdc-14/fzy-1*	*cki-1*
219	0	0	0	1	0	1	1	1
16	0	1	0	1	0	1	1	1
12	0	0	0	0	0	1	1	1
5	0	1	0	0	0	1	1	1
4	0	0	0	0	0	0	0	0

Each row represents an attractor. The first column is the basin size of each attractor. The other 8 columns show the node's state of the attractor.

### Biological pathway in cell-cycle progression

For the largest attractor, four nodes ('*lin-35/efl-1/dpl-1*', '*fzr-1*', '*cdc-14/fzy-1*' and '*cki-1*') were turned on, indicating that cells exited from the M phase. They were at the M/S transition state due to the functions of those genes or proteins (see Methods). At this stable state, cells were waiting to start the cell-cycle process, similar to the checkpoint mechanism in yeast cell-cycle networks [2]. When node '*cdk-2*/cyclinE' turned on in the simulation, cell entered into the S phase. To study how the cell cycle progressed when node '*cdk-2*/cyclinE' was turned on in the simulation, we ran the simulation by the update rules to study the cell-cycle progression in *C. elegans *early embryonic cells. In Table 3, it showed that the cell cycle began at time point 1 where the cell was in the S phase. At time point 2, the node '*cdk-2*/cyclinE' was turned off, indicating the cell exited the S phase. Then, the cell entered the S/M transition state until time point 5 where the node '*cdk-1*/cyclinB' was turned on. Finally, the node '*cdk-1*/cyclinB' was turned off after 3 time points which represented the cell exited the M phase. Interestingly, the state of the cell at time point 8 was the largest attractor in the network model. Therefore, the cell returned to its most stable state.

**Table 3:** Temporal evolution of network states in network model.

Time	*cdk-2*/cyclinE	*cdc-25.1*	*cul-1/lin-23*	*lin-35/efl-1/dpl-1*	*cdk-1*/cyclinB	*fzr-1*	*cdc-14/fzy-1*	*cki-1*	Phase
1	1	0	0	1	0	1	1	1	S
2	0	0	1	0	0	1	0	1	S/M
3	0	0	0	1	0	0	0	1	S/M
4	0	0	0	1	0	0	0	0	S/M
5	0	0	0	1	1	0	0	0	M
6	0	0	0	1	1	0	1	0	M
7	0	0	0	1	1	1	1	1	M
8	0	0	0	1	0	1	1	1	M/S

The numbers in the first column do not reflect the actual time duration. The last column shows which phases the cells stay in.

**Figure 2:**
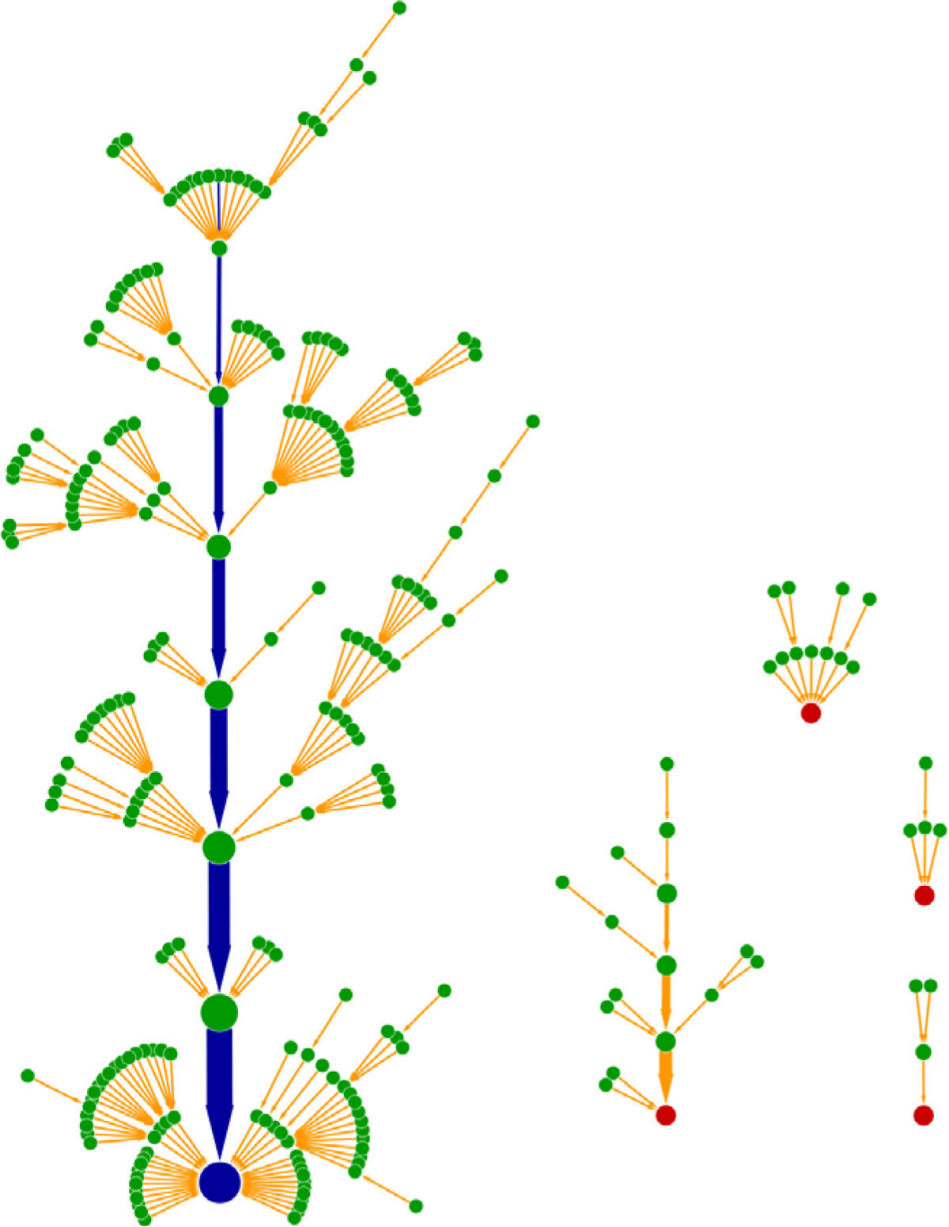
**Dynamical trajectories of 256 initial states flowing to fixed points**. Each node represents an initial state. Red and blue nodes are five attractors, where the blue one is the largest attractor. Arrows denote the transitions between states. The blue arrows represent the 8 steps in Table 3.

In Figure 2, the dynamic trajectories of all 256 initial states converged to the attractors. Each node represented an initial state. The red and blue nodes represented five attractors, where the blue node denoted the largest attractor. Each arrow indicated a dynamic change from one state to another. The blue arrows represented the cell-cycle sequential events (biological pathway) in Table 3. The biological pathway was a very stable trajectory where other dynamic process of the states would converge to.

### Comparison with random networks

**Figure 3:**
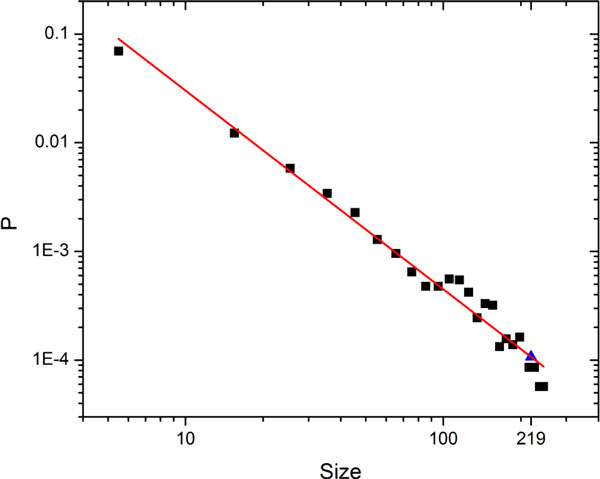
**Attractor basin size distribution of random networks**. The basin sizes are calculated from 1000 same size random networks. P is the probability of the basin size. The blue triangle point represents the attractor which owns same basin size of the largest attractor of the *C. elegans *network.

To study how likely the largest attractor in *C. elegans *network could arise by chance, we analyzed 1000 random networks with the same size. The numbers of nodes, activation edges and repression edges of the random network were same as in the *C. elegans *network. We obtained the following findings from the simulation results. First, there were more attractors (17.57) existed in the random networks than in the *C. elegans *network (5). Second, in random networks, the basin size of the largest attractor (average 105.56) was smaller than that in the *C. elegans *network (219). Thus, a power law was followed by the distribution of the basin size of attractors in the random networks (Figure 3). In 1000 random networks, only 1.1% attractors own a larger basin size than that in the *C. elegans *network (219).

### Network perturbations

**Figure 4:**
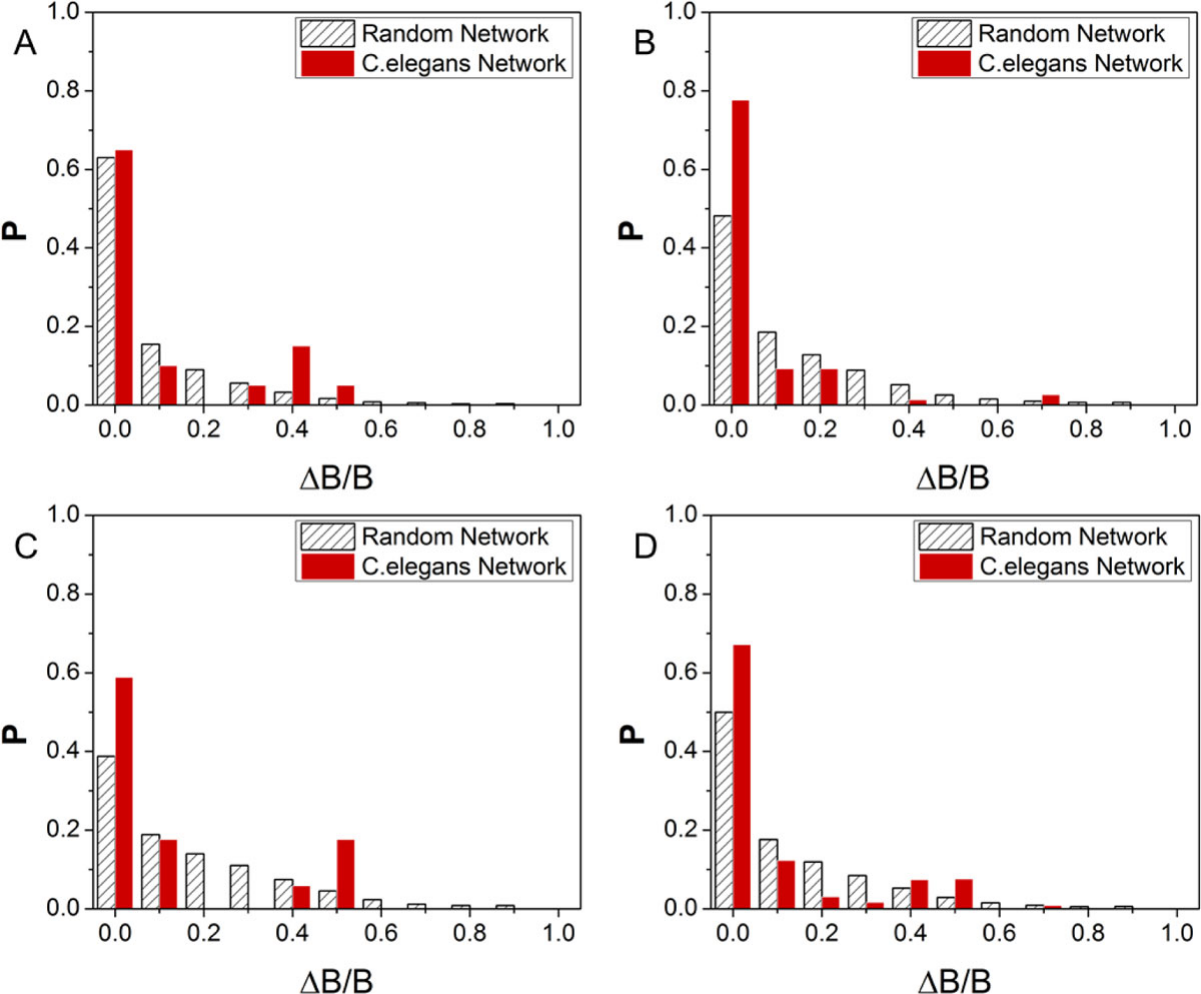
**The histogram of the relative changes of basin size**. The change of the largest attractor's basin size under several network perturbations: (A) deletion, (B) addition, (C) switching and (D) average of A to C. The histogram is generated from the *C. elegans *network and 1000 same size random networks. P is the probability of ΔB/B.

The basin size of attractors in a network is an important index to reflect the stability of the network. The changes (ΔB/B) of the largest attractor's basin size is used to measure the network robustness. Several methods were used to test the robustness of the *C. elegans *network and the random networks under perturbations. The perturbations included deleting an interaction, adding an (activating or repressing) interaction, or switching an interaction. The value of ΔB/B was measured as the results of perturbations. The distribution of ΔB/B under several perturbations, both in the *C. elegans *network and the random networks, were shown in Figure 4. The result showed that the changes of the largest attractor's basin size (ΔB/B) was small. They were close to 0 for most perturbations. There was also a larger probability for the *C. elegans *network than for the random network that the basin size of the largest attractor remained unchanged (Figure 4D). Therefore, the *C. elegans *early embryonic cell cycles network possessed a high homeostatic stability because the basin size of the largest attractor would not change significantly under perturbations [23]. Such high robustness of the *C. elegans *early embryonic cell cycle network was due to the topological structure (nodes and edges) of the regulatory network.

### Comparison with RNAi gene knock down experiment

**Figure 5:**
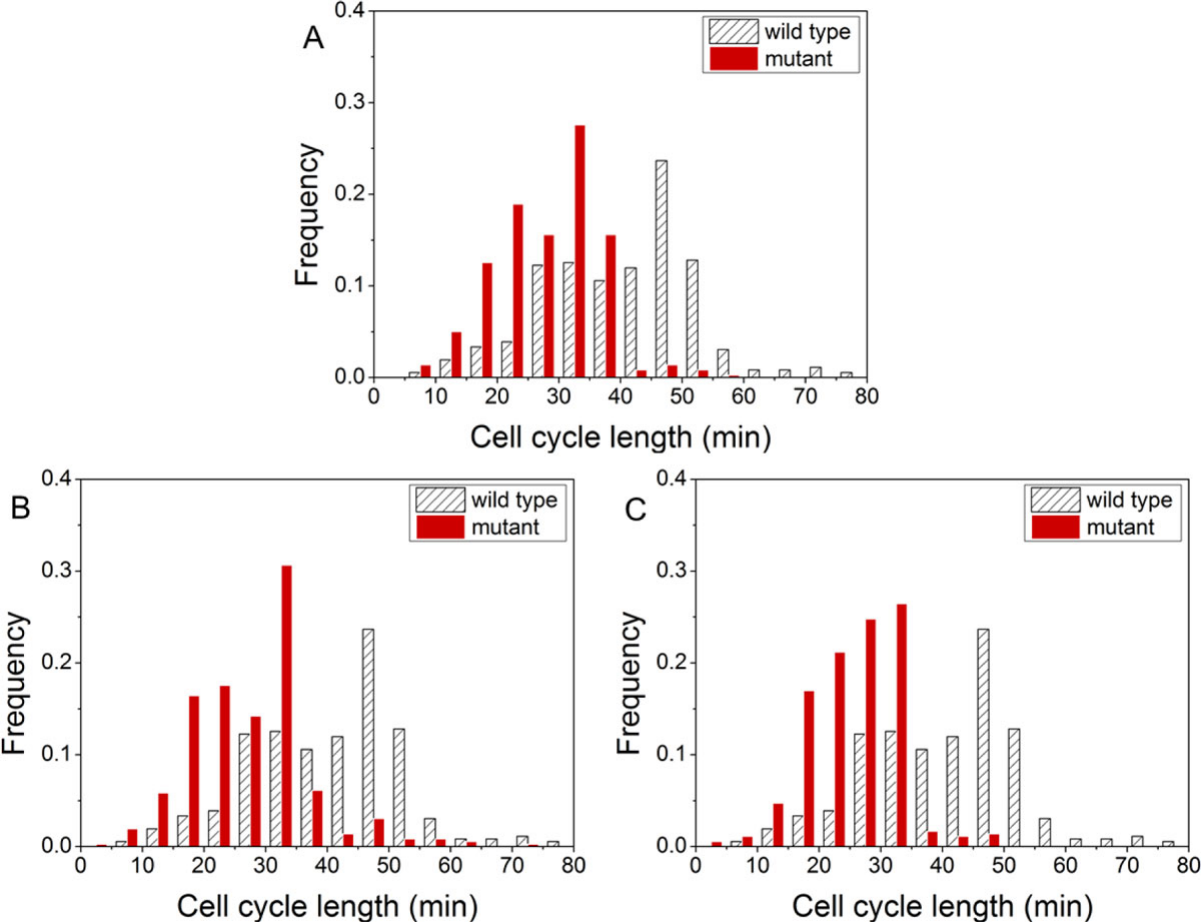
**The histogram of cell-cycle lengths**. The cell-cycle lengths are computed for both the wild type and the mutants: (A) gene *cki-1 *knock down, (B) gene *efl-1 *knock down and (C) gene *cdc-14 *knock down. The results are obtained from the RNAi gene knock down data (see supplementary data file).

Next, we used the RNAi gene knock down experiment data from our biology laboratory (see Methods) to test our network under gene knock down perturbations. In the experiments, genes *efl-1*, *cdc-14*, and *cki-1 *were knocked down. Cells divided faster in mutant than in the wild type (Figure 5). In the mutant, the average cell-cycle lengths were 27.7, 25.4, and 27.1 mins with *cki-1*, *cdc-14 *and *efl-1 *gene knock down respectively. The cell-cycle lengths in the mutants were shorter than that in the wild type (40.3 mins). This could be attributed to the functions of these genes: *efl-1 *repressed the activity of *cdk-2*/cyclinE complex, and *cki-1 *and *cdc-14 *inhibited the expression of *cdk-1*/cyclinB. In our network model, we set the weights of these three nodes to 0 in turn in each simulation, indicating the genes were knocked down. During the updates, the node that represented the knocked down genes would not affect other interacting nodes. We used '*cdc-14 *test', '*efl-1 *test' and '*cki-1 *test' to represent the weights of node '*cdc-14/fzy-1*', node '*lin-35/efl-1/dpl-1*' and node '*cki-1*' to 0 respectively. The number of attractors decreased from 5 to 4 and 5 to 3 respectively in '*cdc-14 *test' and '*efl-1 *test'. The network became more stable when the number of attractors decreased, meaning that more initial states would converge to the same attractor. Moreover, a shorter (seven time points) biological pathway was observed in '*cki-1 *test' (Table 5). We have shown the biological pathway in Table 3, which possessed eight time points for an entire cell cycle. The node '*cki-1*' was always inactive during the simulation, leading to the loss of inactivation of the node '*cki-1*' (Steps 3 and 4 in Table 3). Therefore, the smaller number of attractors and the shorter biological pathway could explain the observation of the cells that divided faster in the knocked down experiment. Thus, the results obtained in our network model in computer simulation matched with the biological experiment results.

**Table 4:** Comparisons between the *C.elegans *early embryonic cell cycles network and other cell-cycle networks in different species

	The *C. elegans *network	**Li, et al. 2004 **[2]	**Davidich, et al. 2008 **[3]	**Yang, et al. 2013 **[5]
**Nodes**	8	11	10	8
**Edges**	21	33	27	21
**Attractors**	5	7	13	5
**Initial states space**	256	2048	1024	256
**Basin size of the biggest attractor**	219	1764	762	184
	85.5%	86%	73%	71.9%

**Table 5:** A biological pathway in '*cki-1 *test'

Time	*cdk-2*/cyclinE	*cdc-25.1*	*cul-1/lin-23*	*lin-35/efl-1/dpl-1*	*cdk-1*/cyclinB	*fzr-1*	*cdc-14/fzy-1*	*cki-1*	Phase
1	1	0	0	1	0	1	1	0	S
2	0	0	1	0	0	1	0	0	S/M
3	0	0	0	1	0	0	0	0	S/M
4	0	0	0	1	1	0	0	0	M
5	0	0	0	1	1	0	1	0	M
6	0	0	0	1	1	1	1	1	M
7	0	0	0	1	0	1	1	1	M/S

## Conclusions and discussion

Modeling the *C. elegans *early embryonic cell cycles is critical for understanding the gene regulation in the cell-cycle process. We have constructed the *C. elegans *early embryonic cell cycle network based on molecular interaction as reported in literatures. We used the Boolean functions to simulate the cell-cycle progression to study the dynamic properties of the network. The *C. elegans *network was then compared with random networks and analyzed under several perturbations to examine the robustness of our network. We have found that the number of attractors of the *C. elegans *network was 5, which was less than one third of the average number of attractors which was 17.57 in 1000 random networks. The largest attractor of the *C. elegans *network contained a basin size of 219, meaning 85.5% of initial states have converged to this attractor (Figure 2). This basin size was more than twice of the average basin size which was 105.56. The basin size from previous cell-cycle network studies were 86% in Li, et al. 2004 [2], 73% in Davidich, et al. 2008 [3], and 71.9% in Yang, et al. 2013 [5]. The basin size (85.5%) of our *C. elegans *early embryonic cell cycles network model is comparable to others (Table 4). Moreover, the main trajectory represented a biological pathway of the entire cell-cycle process. This trajectory simulated the cell cycle starting from the most stable state and finally returning to the original stable state (Table 3). The basin size of the largest attractor did not change under various perturbations. The probability of unchanged basin size of the largest attractor was higher in the *C. elegans *network than in the random networks. In addition, RNAi gene knock down experiment results could be interpreted using our network model. All the above results showed that network model proposed here will be useful for the study of the *C. elegans *early embryonic cell cycles.

In our model, the update rule we used is a type of synchronous model. Synchronous Boolean model for biological control has been used since 1969 in Kauffman's work [7]. In synchronous update rule, all variables at the present time point are able to update their values simultaneously for the next time point. Practically, there is a variety of timescales for different genes/proteins which switch their states. For example, the reaction speeds are different among interactions [24]. A node value changes after several time points rather than at the next time point. Therefore, in contrast, a continuous model, or asynchronous methods, will yield a more realistic temporal description of a biological system [25]. For example, in Mangla, et al. 2010 [24], a timing robustness model is applied, which is asynchronous, to analyze the previous networks in yeast cell-cycle networks. Asynchronous methods will be further studied for a variety of timescales for different genes/proteins.

The topology of a network will determine its dynamic consequence. The topological structure demonstrates how the nodes and their interactions construct the network. Therefore, regulators and their interactions play a key role in the cell-cycle network construction. A lot of regulators participate in cell-cycle regulation in *C. elegans*. However, some interactions between regulators, which participate in G1 and G2 phases, are still not well understood. Details of regulators and their interactions are needed in the future to construct a more sophisticated network and to precisely describe the cell cycle process.

## Competing interests

The authors declare that they have no competing interests.

## Authors' contributions

XH carried out the network construction and simulation work and drafted the paper. LC and HC developed experiment data processing and analysis tools. LLHC, ZZ and HY initiated the project and helped with the writing of the paper. ZZ's group obtained the RNAi gene knock down experiment data. All authors read and approved the final manuscript.

## Declarations

This article is published as part of a supplement. Hong Kong Research Grants Council (RGC) (Project HKBU5/CRF/11G) has provided the funding for publication of the article.

This article has been published as part of *BioMedical Engineering OnLine *Volume 12 Supplement 1, 2013: Selected articles from the 35th Annual International Conference of the IEEE Engineering in Medicine and Biology Society: Workshop on Current Challenging Image Analysis and Information Processing in Life Sciences. The full contents of the supplement are available online at http://www.biomedical-engineering-online.com/supplement/12/S1
